# Will COVID-19 become the next neglected tropical disease?

**DOI:** 10.1371/journal.pntd.0008271

**Published:** 2020-04-10

**Authors:** Peter J. Hotez, Maria E. Bottazzi, Sunit K. Singh, Paul J. Brindley, Shaden Kamhawi

**Affiliations:** 1 Departments of Pediatrics and Molecular Virology & Microbiology, Texas Children’s Hospital Center for Vaccine Development, National School of Tropical Medicine, Baylor College of Medicine, Houston, Texas, United States of America; 2 Department of Biology, Baylor University, Waco, Texas, United States of America; 3 Hagler Institute for Advanced Study at Texas A&M University, Texas, United States of America; 4 James A Baker III Institute of Public Policy, Rice University, Houston, Texas, United States of America; 5 Scowcroft Institute of International Affairs, Bush School of Government and Public Service, Texas A&M University, Texas, United States of America; 6 Molecular Biology Unit, Faculty of Medicine, Institute of Medical Sciences, Banaras Hindu University, Varanasi, India; 7 Department of Microbiology, Immunology, and Tropical Medicine, George Washington University School of Medicine, Washington D.C., United States of America; 8 Vector Molecular Biology Section, Laboratory of Malaria and Vector Research, National Institute of Allergy and Infectious Diseases, National Institutes of Health, Bethesda, Maryland, United States of America; New York Blood Center, UNITED STATES

The daily World Health Organization (WHO) Coronavirus Situation Reports highlight the rapid spread of COVID-19 across Europe, the United States, and many of the advanced nations in East Asia [1]. Currently, the major low- and middle-income nations such as Bangladesh, Brazil, Democratic Republic of Congo, India, Indonesia, Mexico, Nigeria, Pakistan, Philippines, and South Africa, as well as Central American and other nations are beginning to report an increase in COVID-19 cases, but the numbers are still relatively small. For example, these highly populated nations now only account for about 1% of the confirmed cases, even though they represent approximately one-third of the global population.

Almost certainly, this situation will shift in the coming weeks and months. We believe there is a high probability the current numbers represent underestimates due to inadequate testing. Surely, lack of access to diagnostic kits comprises one of the many components of weak health systems in resource-poor nations.

Beyond testing, it may turn out that the seasonal nature of some respiratory virus pathogens might also extend to the SARS CoV2. This means that cases would decline with warming temperatures, independent of control efforts. However, this would also indicate that though COVID-19 might be peaking in the northern hemisphere this winter and spring, there is a real possibility that it will advance into tropical countries and the Southern Hemisphere later this year. In such case, some of the nations highlighted above might be vulnerable to the next wave of SARS CoV2 dissemination. SARS CoV2 is a new virus pathogen, and we need to appropriately assess its behavior over the span of a full year.

If SARS CoV2 becomes a major respiratory virus pathogen in resource-poor countries of the tropics and subtropics, we might envision unprecedented levels of global morbidity and mortality. We have already seen how even strong health systems in New York and northern Italy quickly became overwhelmed, and one can only imagine the terrible consequences of this virus in the poorest reaches of Africa, Asia, and Latin America. Based on the levels of illness we have seen to date in the Northern Hemisphere, we are especially worried about the fate of thousands of dedicated doctors, nurses, and other health care providers.

Accordingly, *PLOS Neglected Tropical Diseases* will consider articles from the community of scientists and public health experts in Asia, Africa, and Latin America now shifting their efforts to combat the COVID-19 pandemic. We therefore welcome original high quality research papers and front matter articles, including viewpoints and editorials.

Our *PLOS Neglected Tropical Disease* editors have just completed an exhaustive assessment of what constitutes a neglected tropical disease in order to keep our scope both relevant and timely [2]. However, as John Lennon once said, “life is what happens to you while you're busy making other plans,” and on that basis we now invite our community of NTD scientists to submit COVID-19 papers on what may become a global health terror on a scale that rivals or even exceeds some of the world’s major neglected tropical diseases.
